# Gambling disorder symptoms among Slovenian gamblers

**DOI:** 10.1093/eurpub/ckac131.481

**Published:** 2022-10-25

**Authors:** Š Selak, M Žmavc, I Makivić

**Affiliations:** National Institute of Public Health, Ljubljana, Slovenia

## Abstract

**Background:**

We are facing a growing popularity of gambling, partly due to the rapid rise of internet related technologies. This growth has been linked to a considerable increase in problem gambling and gambling disorder, which has been an established non-substance addiction. The aim of our study was to assess the prevalence rate of gambling disorder among the Slovenian population.

**Methods:**

National Survey on the Use of Tobacco, Alcohol and Other Drugs was conducted in 2018 on a nationally representative sample (n = 16,000; age: 15-64 years), using mixed-mode data collection (CAWI and CAPI). Responses were obtained from 9,161 respondents, of those 18.4% (n = 1,686) reported to have gambled in the last 12 months (i.e. gamblers). Gambling disorder symptoms were assessed using Berlin Inventory of Gambling Behavior - Screening, consisting of 14 items with a yes/no response option, which measure 9 gambling disorder criteria (DSM-V). Participants who reported at least 5 out of 9 gambling disorder criteria were classified as disordered.

**Results:**

Data shows that 4.3% of Slovenian gamblers met the criteria for gambling disorder. Significantly higher shares were observed among men (5.9%; compared to 1.2% of women) and among younger generations (highest shares among 15-19 year olds (19.4%) and 20-24 year olds (14.5%)). The same goes for money spent for gambling. Namely, males (4.4% of men; compared to 0.6% of women) and younger generations (highest shares among 20-24 year olds (9.2%) and 30-34 year-olds (6.9%)) were more likely to spend more than 100 EUR in a day for gambling.

**Conclusions:**

Data obtained in the present study indicates the extent of gambling disorder symptomatology and highlights key demographic groups with risk for gambling disorder. These findings are consistent with previous comparable studies and provide a basis for tailored public health measures in Slovenia with an emphasis on early intervention.

**Key messages:**

• 4.3% of Slovenian residents, who engaged in gambling in the last 12 months met the criteria for gambling disorder.

• Males and younger generations were more likely to report gambling disorder symptoms and spent more money on gambling. The data shows the need for tailored public health measures.

